# Association of frailty with mortality in cancer survivors: results from NHANES 1999–2018

**DOI:** 10.1038/s41598-023-50019-1

**Published:** 2024-01-18

**Authors:** Chongya Zhai, Luxi Yin, Jiaying Shen, Jie Dong, Yu Zheng, Hongming Pan, Weidong Han

**Affiliations:** 1 Department of Medical Oncology, Sir Run Run Shaw Hospital, College of Medicine, Zhejiang University, 3 East Qingchun Road, Hangzhou, 310000 Zhejiang People’s Republic of China; 2 Department of Medical Oncology, Shaoxing Campus, Sir Run Run Shaw Hospital, College of Medicine, Zhejiang University, Shaoxing, China

**Keywords:** Oncology, Risk factors

## Abstract

Cancer survivors are vulnerable to frailty. While few studies have focused on the association of frailty with mortality risk among cancer survivors, the current study aimed to reveal this association. In this cohort study, 4723 cancer survivors were enrolled from the National Health and Nutrition Examination Surveys (NHANES, 1999–2018). Frailty status was quantified using the 53-item frailty index. Death outcomes were linked to National Death Index mortality data (as of December 31, 2019). Cox proportional hazard models were used to estimate HRs (95% CIs). The median (IQR) frailty score was 0.190 (0.132, 0.277). During the median follow-up of 6.7 years, 1775 all-cause deaths (including 581 cancer deaths and 385 cardiac deaths) were documented. Compared to the lowest tertile of frailty scores, the adjusted HRs (95% CIs) for the highest tertile were 2.698 (2.224, 3.272) for all-cause mortality (*P* trend < 0.001), 2.145 (1.547, 2.973) for cancer mortality (*P* trend < 0.001), and 3.735 (2.231, 6.251) for cardiac mortality (*P* trend < 0.001). Moreover, a positive dose‒response association between the frailty score and mortality risk was determined. Each per-unit increase in the frailty score (natural logarithm transformed) was found to increase all-cause mortality by 159% (*P* < 0.001), cancer mortality by 103% (*P* < 0.001), and cardiac mortality by 256% (*P* < 0.001). A consistent result was shown when stratifying by age, sex, race, body mass index, and type of cancer. This study suggested that the frailty index was positively associated with all-cause mortality and cause-specific mortality (including cancer and cardiac deaths) among cancer survivors.

## Introduction

Cancer is one of the leading causes of death worldwide and imposes an enormous economic burden on public health systems^1,2^. Although cancer survival rates have improved substantially over the past few decades, the 5-year survival rate for cancer patients remains low at approximately 68.1%^3^. In terms of socioeconomic burden, cancer survivors spend an average of $3000–$4000 more on medical expenses per year than noncancer patients^4^. Therefore, it is important to identify modifiable risk factors in cancer patients to improve their prognosis and reduce the corresponding socioeconomic burden.

Frailty, which is defined as a clinical syndrome of reduced reserve and resistance to stressors or an accumulation of conditions causing vulnerability, could be a potential focus^5–7^. Cancer survivors are more likely to suffer from frailty^8,9^, which makes them vulnerable to stressful events such as infection, chemotherapy, radiation, or surgery because of the diminished ability to resist stressors. More importantly, frailty could be reversible or improved by adequate nutritional supplementation^10^ and appropriate physical exercise^11^, making it a potential intervention target.

Two main frailty models have been developed based on conceptual definitions, including the phenotype model and the frailty model. The phenotypic approach was first documented in the Cardiovascular Health Study^12^, which defined frailty as meeting 3 of 5 physical criteria or more. The frailty index model was constructed based on a more comprehensive clinical judgment by counting various clinical deficits, including underlying diseases, psychosocial status, and other signs and symptoms of geriatric patients^13^. In contrast, several head-to-head comparisons have demonstrated that the frailty index is superior to the frailty phenotype in predicting the risk of negative health outcomes^14,15^. However, few studies have focused on the relationship between the frailty index and mortality risk among cancer survivors.

Therefore, we conducted this cohort study to investigate the association of the frailty index with all-cause and cause-specific mortality in cancer survivors.

## Result

The baseline characteristics of 4723 participants according to frailty score tertile are summarized in Table 1. The median frailty score was 0.190 (IQR 0.132–0.277). Cancer survivors with higher frailty scores were more likely to be female and smokers, have a higher BMI, diabetes, and hypertension, participate in no leisure-time physical activity, and have higher education levels.

**Table 1:** Baseline characteristics among cancer patients according to frailty score.

Variables	Total	Frailty score			*P* value
		Tertile 1 [0.0345,0.152)	Tertile 2 [0.1516,0.240)	Tertile 3 [0.2403,0.767]	
Number of patients	4723	1575	1574	1574	
Frailty score	0.20 ± 0.00	0.11 ± 0.00	0.19 ± 0.00	0.34 ± 0.00	< 0.001
Age, years	62.19 ± 0.31	60.19 ± 0.53	62.41 ± 0.46	64.94 ± 0.54	< 0.001
Body mass index, kg/m^2^	28.72 ± 0.13	27.29 ± 0.19	28.97 ± 0.20	30.56 ± 0.25	< 0.001
Sex (%)					0.010
Female	2536	806 (57.49)	810 (56.20)	920 (63.60)	
Male	2187	769 (42.51)	764 (43.80)	654 (36.40)	
Race (%)					< 0.001
Non-Hispanic White	3182	1157 (89.91)	1039 (84.39)	986 (79.02)	
Non-Hispanic Black	709	181 (3.50)	252 (6.71)	276 (9.15)	
Other	832	237 (6.60)	283 (8.90)	312 (11.83)	
Education level (%)					< 0.001
Less than high school	519	103 (2.35)	164 (5.59)	252 (9.67)	
High school	1695	478 (26.09)	580 (34.01)	637 (38.13)	
College or above	2509	994 (71.56)	830 (60.40)	685 (52.20)	
Poverty income ratio (%)					< 0.001
≤ 1.0	726	139 (5.37)	209 (9.83)	378 (19.57)	
1.0–3.0	2060	569 (26.75)	686 (34.13)	805 (46.54)	
> 3.0	1937	867 (67.88)	679 (56.04)	391 (33.90)	
Smoking status (%)					< 0.001
Never	2100	772 (50.06)	699 (44.23)	629 (38.90)	
Former	1886	592 (34.97)	646 (40.06)	648 (39.17)	
Current	737	211 (14.97)	229 (15.71)	297 (21.94)	
Drinking status (%)					< 0.001
None	2292	604 (26.87)	718 (39.19)	970 (52.35)	
Mild	1251	495 (34.86)	444 (30.00)	312 (24.49)	
Heavy	1180	476 (38.26)	412 (30.81)	292 (23.16)	
Leisure-time physical activity (%)					< 0.001
No	3150	843 (46.35)	1032 (61.21)	1275 (77.04)	
Yes	1573	732 (53.65)	542 (38.79)	299 (22.96)	
Diabetes mellitus					< 0.001
No	3525	1402 (90.54)	1182 (77.27)	941 (63.33)	
Yes	1198	173 (9.46)	392 (22.73)	633 (36.67)	
Hypertension					< 0.001
No	1640	804 (57.54)	485 (35.88)	351 (23.68)	
Yes	3083	771 (42.46)	1089 (64.12)	1223 (76.32)	
Hyperlipidemia					0.200
No	1295	439 (23.93)	411 (22.36)	445 (20.16)	
Yes	3428	1136 (76.07)	1163 (77.64)	1129 (79.84)	
Type of cancer					< 0.001
Skin and soft tissue	1083	487 (38.63)	334 (28.36)	262 (22.15)	
Urinary system	996	330 (12.16)	347 (14.79)	319 (15.21)	
Breast	792	249 (16.10)	268 (16.95)	275 (18.24)	
Genital system	678	195 (14.25)	227 (16.53)	256 (18.54)	
Digestive system	475	107 (5.02)	152 (6.89)	216 (11.29)	
Other	699	207 (13.85)	246 (16.48)	246 (14.57)	

Continuous variables are shown as weighted means ± standard errors. Categorical variables are shown as unweighted counts (weighted percentages). All estimates accounted for complex survey designs.

**Figure 1:**
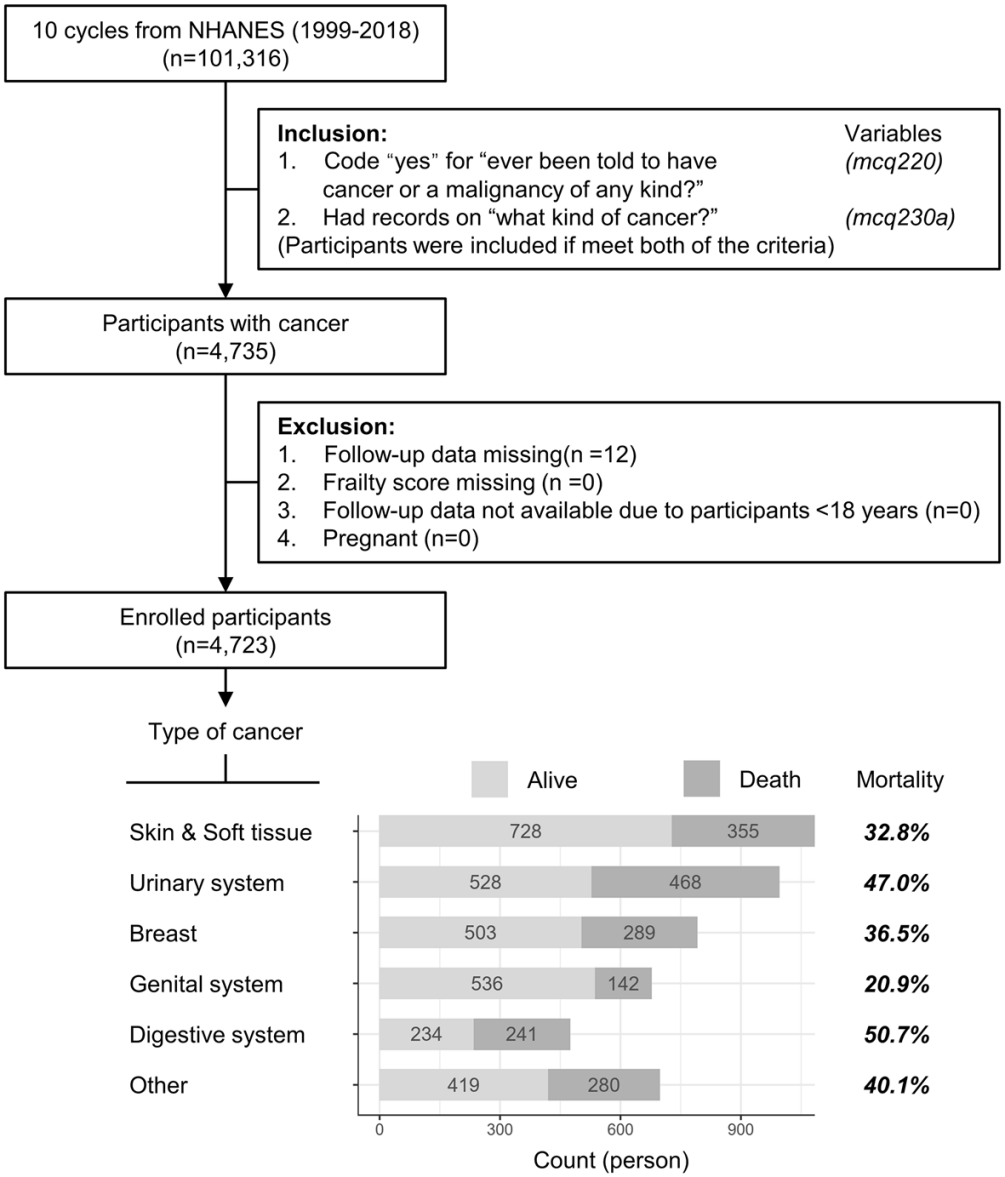
Flowchart and population composition according to type of cancer.

During an average follow-up of 6.7 years, 1775 all-cause deaths were documented, including 581 cancer deaths and 385 cardiac deaths. According to the type of cancer, the number of cancer survivors (mortality rate) was as follows: 1083 (32.8%) survivors of skin or soft tissue cancer, 996 (47%) survivors of urinary system cancer, 792 (36.5%) survivors of breast cancer, 678 (20.9%) survivors of genital system cancer, and 475 (40.7%) survivors of digestive system cancer (Fig. 1).

**Figure 2:**
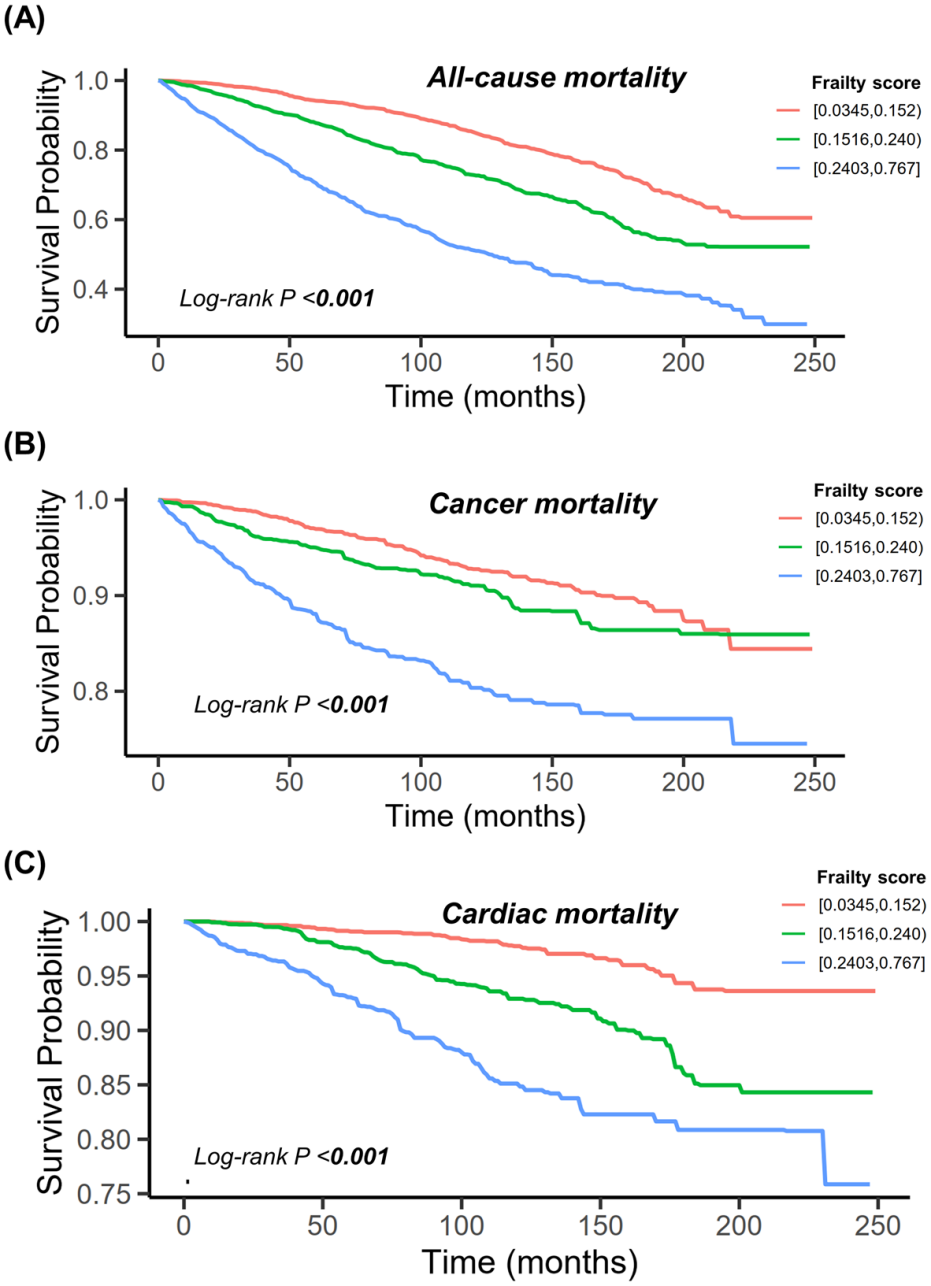
Kaplan‒Meier survival curve of all-cause mortality (**A**), cancer mortality (**B**), and cardiac mortality (**C**) according to frailty score tertiles among patients with cancer.

Kaplan‒Meier survival analyses showed that all-cause mortality (Fig. 2A), cancer mortality (Fig. 2B), and cardiac mortality (Fig. 2C) significantly differed according to frailty score categories (log-rank test, all *P* values < 0.001).

In multivariable Cox regression, adjusted HRs (95% CIs) across frailty categories were 1.000 (reference), 1.459 (1.209, 1.761), and 2.698 (2.224, 3.272) for all-cause mortality (*P* trend < 0.001), 1.000 (reference), 1.138 (0.853, 1.517) and 2.145 (1.547, 2.973) for cancer mortality (*P* trend < 0.001), and 1 (reference), 2.046 (1.288, 3.248) and 3.735 (2.231, 6.251) for cardiac mortality (*P* trend < 0.001) (Table 2). Each per-unit increase in the frailty score (natural logarithm transformed) was found to increase all-cause mortality by 159% (*P* < 0.001), cancer mortality by 103% (*P* < 0.001), and cardiac mortality by 256% (*P* < 0.001) (Table 2).

**Table 2:** HRs (95% CIs) for all-cause and cause-specific mortality among cancer patients according to frailty score.

Characteristics	Frailty score			P-trend	Per-unit increase in the frailty score (Ln-transformed)
	Tertile 1 [0.0345,0.152)	Tertile 2 [0.1516,0.240)	Tertile 3 [0.2403,0.767]		
All-cause mortality					
No. deaths/total (%)	402/1575 (25.5)	559/1574 (35.5)	814/1574 (51.7)		1775/4723 (37.6)
Model 1	1 (reference)	1.602 (1.349, 1.903)	3.392 (2.859, 4.025)	< 0.001	3.086 (2.675, 3.560)
Model 2	1 (reference)	1.522 (1.280, 1.810)	2.862 (2.380, 3.442)	< 0.001	2.671 (2.309, 3.091)
Model 3	1 (reference)	1.459 (1.209, 1.761)	2.698 (2.224, 3.272)	< 0.001	2.593 (2.239, 3.002)
Cancer mortality					
No. deaths/total (%)	133/1575 (8.4)	186/1574 (11.8)	262/1574 (16.6)		581/4723 (12.3)
Model 1	1 (reference)	1.262 (0.961, 1.658)	2.782 (2.078, 3.725)	< 0.001	2.505 (1.944, 3.227)
Model 2	1 (reference)	1.172 (0.897, 1.532)	2.222 (1.637, 3.018)	< 0.001	2.059 (1.586, 2.673)
Model 3	1 (reference)	1.138 (0.853, 1.517)	2.145 (1.547, 2.973)	< 0.001	2.029 (1.536, 2.679)
Cardiac mortality					
No. deaths/total (%)	68/1575 (4.3)	127/1574 (8.1)	190/1574 (12.1)		385/4723 (8.2)
Model 1	1 (reference)	2.415 (1.572, 3.710)	5.237 (3.290, 8.336)	< 0.001	4.501 (3.182, 6.365)
Model 2	1 (reference)	2.232 (1.443, 3.454)	4.302 (2.574, 7.188)	< 0.001	3.948 (2.666, 5.848)
Model 3	1 (reference)	2.046 (1.288, 3.248)	3.735 (2.231, 6.251)	< 0.001	3.557 (2.396, 5.282)

HR (95% CI) was estimated by the Cox proportional hazards model and accounted for the sample weights. According to the ICD-10 criteria, cardiac mortality was defined as I00-I09, I11, I13, and I20-I51, and cancer mortality was defined as C00-C97. Model 1 was adjusted for age (continuous), race (non-Hispanic white, non-Hispanic black, or other), and sex (male or female). Model 2 was additionally adjusted for BMI (< 20, 20–24, 25–29, or ≥ 30 kg/m^2^), educational attainment (below high school, high school, or college or above), alcohol consumption (none, mild, or heavy), cigarette consumption (never, former, or current), poverty income ratio (≤ 1, 1–3, or > 3), and leisure-time physical activity (no, yes). Model 3 was additionally adjusted for diabetes (no or yes), hyperlipidemia (no or yes), hypertension (no or yes), and the type of cancer (skin and soft tissue, urinary system, breast, genital system, digestive system, and other).

**Figure 3:**
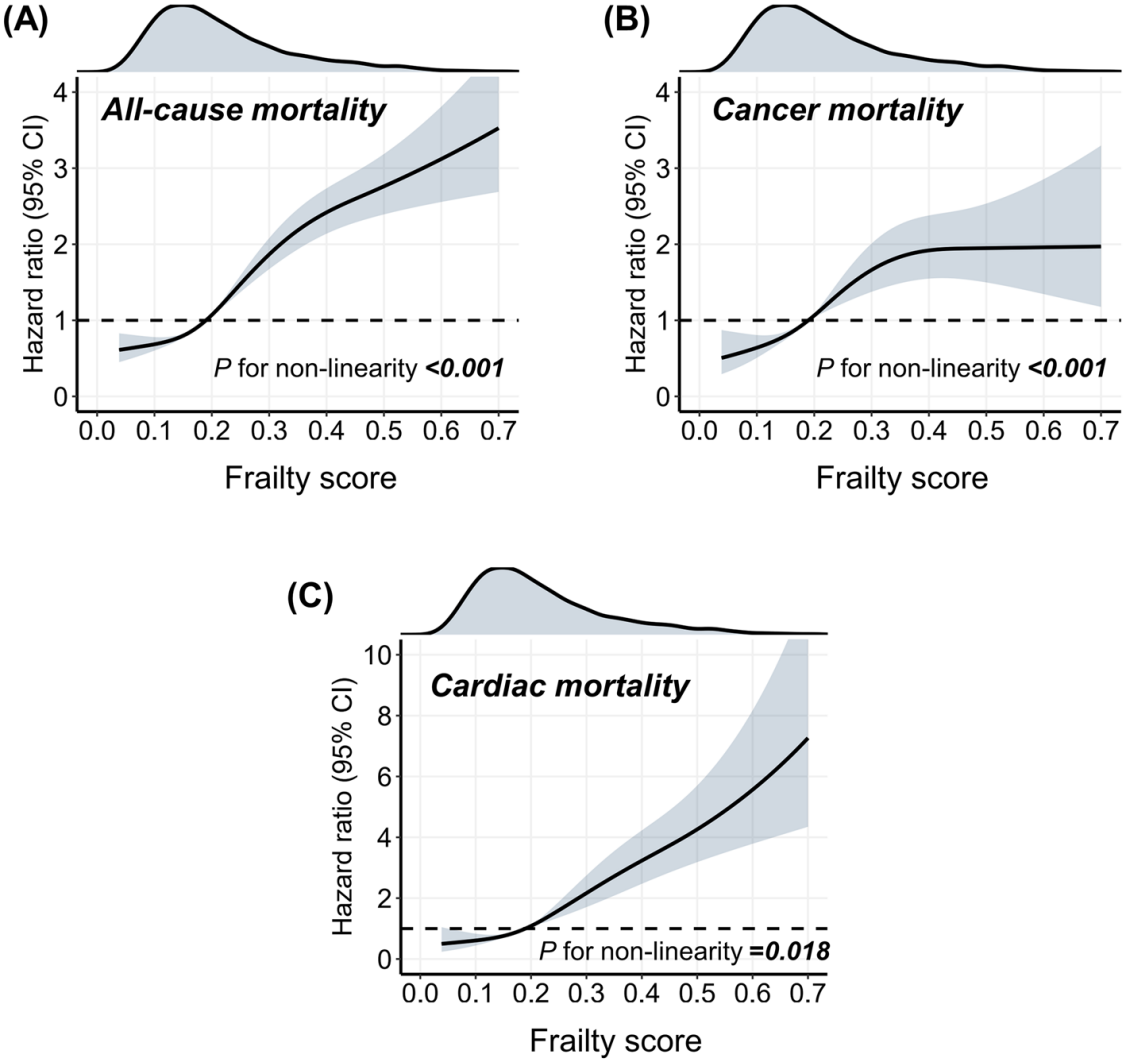
The dose‒response association of the frailty score with all-cause mortality (**A**), cancer mortality (**B**), and cardiac mortality (**C**) among patients with cancer. The dose‒response association of the continuous frailty score with mortality risk was visualized by the restricted cubic spline model. Four knots of the spline model were determined at specific distribution percentiles (5%, 35%, 65%, and 95%). The spline model was adjusted for consistent confounding factors, including age, race, sex, BMI, educational attainment, alcohol consumption, cigarette consumption, poverty income ratio, leisure-time physical activity, diabetes, hyperlipidemia, hypertension, and the type of cancer (skin and soft tissue, urinary system, breast, genital system, digestive system, and other).

In Fig. 3, spline analysis indicated a general positive association between the frailty score and various mortality risks. For frailty scores greater than approximately 0.2, the risk of various types of mortality increased rapidly. Particularly, for cancer death, the mortality risk became flat when the frailty score was greater than approximately 0.4.

**Figure 4:**
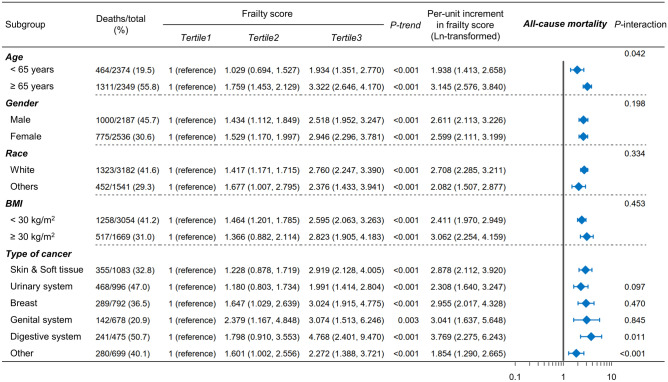
Subgroup analyses of the association of the frailty score with all-cause mortality among patients with cancer. HR (95% CI) was assessed by the Cox proportional hazards model. The model was adjusted for covariates, including age, race, sex, BMI, educational attainment, alcohol consumption, cigarette consumption, poverty income ratio, leisure-time physical activity, diabetes, hyperlipidemia, hypertension, and the type of cancer (except for the stratified variables themselves).

In subgroup analyses (Figs. 4, S1–2), consistent results were observed when survivors were stratified by age (≤ 65, > 65 y), sex (male, female), race (white, others), BMI (< 30, ≥ 30 kg/m^2^) and type of cancer (skin and soft tissue, urinary system, breast, genital system, digestive system, and others). For cancer mortality (Fig. S1), there was no significant interaction between the frailty score and the type of cancer (all *P* > 0.05).

In sensitivity analyses, the association of interest remained largely unchanged in the cubic spline models after excluding survivors with frailty scores outside the 5th and 95th percentiles (Fig. S3). In terms of other cause-specific mortality and residual mortality, the Kaplan‒Meier curves still significantly differed according to frailty score categories (Fig. S4). In multivariable Cox regression, adjusted HRs (95% CIs) across frailty categories were 1.000 (reference), 1.350 (0.882, 2.067), and 2.137 (1.419, 3.219) for other cause-specific mortality (*P* trend < 0.001) and 1.000 (reference), 1.627 (1.107, 2.391), and 3.528 (2.393, 5.199) for residual mortality (*P* trend < 0.001) (Table S4). When additionally adjusting for detailed cancer classifications (29 types, refer to Table S1), the association between frailty and mortality remained consistent (Table S5).

## Discussion

This study investigated the association of frailty status with all-cause and cause-specific mortality in 4723 cancer survivors. During the median follow-up of 6.7 years, a per-unit increase in the frailty score (Ln-transformed) was associated with a 2.59-fold increased risk of all-cause mortality. Consistently, this positive association was also verified in cause-specific mortality (including cancer and cardiac deaths) and demonstrated across the stratified population (including different types of cancer).

The frailty index scoring system was first proposed by the Canadian Study of Health and Aging and defined as the ratio of the cumulative deficits present to the number of total deficits considered^13^. Cancer survivors are at higher risk of frailty, which increases with age^8,9^. In almost every study, the associations between frailty and poor outcomes are consistent regardless of measures, with frail individuals at higher risk of chronic disease and death^16–18^.

Frailty status has been found to result in some negative health outcomes. Shahrokni et al., in a cohort of 1137 patients with cancer, reported that higher frailty scores were significantly associated with a longer postsurgical length of stay and a higher risk of intensive care unit admission and 1-year mortality^19^. Williams et al. evaluated the associations between frailty and long-term functional outcomes and found that frailty increased the risk of hospital admission by 2.5-fold and long-term care admission by 1.9-fold^20^. A study evaluating the association between frailty status and quality of life over time in patients after colorectal cancer surgery showed that quality of life scores were significantly lower among survivors with frailty than among those without frailty at baseline and at 3 and 18 months after surgery^21^.

In addition, frailty status has also been found to increase mortality risk in specific populations. Brown et al. evaluated associations between frailty and survival time among elderly cancer survivors and found a median survival of 13.9 years among nonfrail survivors and 2.5 years among frail survivors^22^. Another study with a cohort of 138 lung cancer patients reported a negative association between frailty and overall survival and a higher number of noncancer-related deaths over 3 years of follow-up^23^. In line with these studies, this study identified a positive association of frailty status with all-cause and cause-specific mortality (including cancer and cardiac deaths) in a heterogeneous cancer population.

There are several possible explanations for the association between frailty and mortality. One widely accepted explanation is that the underlying causes of frailty in cancer survivors, such as aging, chronic inflammation, or poor nutritional status, are also strongly associated with mortality risk^24^. Additionally, studies have shown that frailty is significantly associated with decreased physical and mental functions, both of which contribute to poor prognosis^25^. The potential biological mechanisms of the association between frailty and mortality should be addressed in detail in future research.

There are several findings of interest in this study. First, among cancer survivors, the frailty index was positively associated not only with cancer mortality but also with mortality from other specific causes (including cardiac mortality). As a composite indicator, the frailty index may also have good predictive performance for other clinical outcomes in cancer survivors. Second, the positive association between the frailty index and cancer mortality was found to be consistent across all types of cancer. The improvement of frailty status could be beneficial regardless of the type of cancer. Third, although an increased frailty index score increased both cancer and cardiac mortality, their dose‒response associations were not identical. For cardiac mortality, the HR increased consistently with an increasing frailty index score. However, for cancer mortality, the HR started to turn flat after the frailty index score was greater than approximately 0.4. This difference may be attributed to the following factors. Our study focused on baseline frailty status as the observed variable, while the survivors' frailty status could vary during the follow-up period. This variability could differ across outcomes. Frailty related to cardiovascular diseases is often associated with a gradual aging process, exhibiting lower variability. On the other hand, frailty associated with cancer may be linked to the rapid progression of the disease, resulting in higher variability. Consequently, we observed that the risk of cancer mortality ceases to increase beyond a certain threshold of baseline frailty.

In this study, we found that women with cancer were more likely to be frail, which is in line with results from other prior studies^26,27^. Women are more likely to have lower body mass and strength than men and are more prone to sarcopenia^28^ because of the greater likelihood of inadequate nutrition intake with age^29^, which makes women more likely to cross the threshold for frailty. In addition, we found that participants with higher frailty scores were more likely to be smokers, have a higher BMI, and participate in no leisure-time physical activity. Many studies have shown that various factors contribute to the development of frail health and its transition, including nutrition intake^30^, personal habits^31^, diseases, and psychological factors^32^. These variable characteristics make frailty a reversible and comprehensive health condition^33^. Therefore, interventions to prevent or improve frailty in cancer survivors should be multifocal. There are currently no published clinical trials in cancer survivors with prevention or improvement of frailty as the primary outcome. Whether the reversal of individual frailty reduces the risk of adverse outcomes requires further investigation.

The current study has a number of strengths. Innovatively, our study focused on community-dwelling cancer survivors with an average age of approximately 62 years, offering unique insights distinct from prior research primarily centered around older, hospital-based populations^8^. The intentional inclusion of younger participants fills a critical gap in frailty research, as evidenced by previous studies imposing age restrictions^34^. Leveraging nationally representative survey data, our findings have broader relevance, contrasting with studies limited to single-center hospital cohorts^34^. These methodological choices underscore the distinctive contribution of our study, providing a valuable perspective on frailty in cancer survivors across diverse demographics and health care settings.

Several limitations should be considered to better understand the results of the present study. First, cancer-related data were derived from participant self-reports, which may be subject to self-reporting bias. To this end, the NHANES adhered to standardized and meticulously controlled procedures to ensure the reliability and integrity of the data. Second, due to limited data, heterogeneity in cancer-related characteristics, such as cancer stage, the time from cancer diagnosis to baseline, and cancer treatments, may not have been fully considered. This may lead to potential imputation bias. To minimize this bias, we adjusted for the detailed cancer classification (29 types) in the analysis. Third, the association of interest was assessed under the assumption of invariance of frailty status, which ignores the variability in frailty. Further longitudinal cohorts that account for variability in frailty are warranted. Finally, due to the nature of observational studies, we were unable to draw causal inferences or assess the potential prognostic benefit of frailty interventions. Further studies are warranted to evaluate frailty interventions.

This study suggested that the frailty index was positively associated with all-cause mortality and cause-specific mortality (including cancer and cardiac deaths) in cancer survivors. The frailty index can be used to predict clinical outcomes in cancer patients and is a potential target for therapeutic intervention. Efforts to identify, manage, and prevent frailty should be implemented for cancer survivors.

## Methods

### Study population and data collection

The data of the current study were extracted from the National Health and Nutrition Examination Surveys (NHANES, 1999–2018). The NHANES is a nationally representative survey conducted by the National Center for Health Statistics^35^. In the current study, participants who met both of the following criteria were included: (1) Those who responded “yes” for “ever been told they have cancer or a malignancy of any kind” (variable mcq220); and (2) Those who had a record for the question “what kind of cancer?” (variable mcq230a). After excluding participants who were < 18 years old (n = 0), were pregnant (n = 0) at baseline, or had no follow-up data or frailty score (n = 1), 4723 self-reported cancer survivors were ultimately included in the current analysis (Fig. 1). The NHANES dataset encompasses a comprehensive classification of cancers, numbering up to 30 types (Table S1). According to the answer to the variable mcq230a, cancer was divided into six types with relatively balanced amounts, including malignant tumors on the skin and soft tissue and cancer of the urinary system, breast, genital system, and digestive system; if participants did not have any of these cancers, they were grouped into the other category. The definition and number of deaths are detailed in Table S2.

To ensure the reliability and integrity of the data, the NHANES questionnaire data collection process adhered to standardized and meticulously controlled procedures. The Computer Assisted Personal Interviewing (CAPI) system played a pivotal role, programmed with built-in consistency checks to minimize data entry errors. Moreover, the CAPI system included an online help screen, providing invaluable assistance to interviewers in accurately defining key terms in the questionnaire. This rigorous quality assurance and control framework underscored our commitment to maintaining high standards of data quality throughout the NHANES, thereby enhancing the credibility and robustness of our findings.

### Frailty index

The frailty index model was calculated based on the standard procedure developed by Searle and colleagues^36^, which was based on a comprehensive geriatric assessment by adding accumulated deficits that covered multiple systems. In this study, seven systems including 53 deficits were introduced to the frailty index model by assigning a value between 0 and 1 according to the severity of the deficit. The seven systems covered the following dimensions: (1) Cognition, containing 1 question about whether the individual suffers from confusion and memory problems; (2) Dependence, containing 20 questions regarding difficulty performing activities of daily living; (3) Depression, containing 7 questions related to depressive symptoms based on the Patient Health Questionnaire (PHQ-9); (4) Comorbidities, containing 13 items regarding self-reported arthritis, thyroid problems, chronic bronchitis, malignant tumor, heart failure, coronary heart diseases, angina, high blood pressure, heart attack, stroke, diabetes, weak kidneys, and urinary leakage; (5) Hospital utilization and access to care, containing 5 questions regarding general health conditions, current health compared with 1 year ago, overnight hospitalization within the last year, the frequency of health care use during the past year, and the number of prescribed medications; 6. Physical performance and anthropometry, containing 1 item about body mass index; and 7. Laboratory values, containing 6 items regarding glycohemoglobin, red blood cell count, hemoglobin, red cell distribution width, lymphocyte percent, and segmented neutrophil percent. The frailty index is demonstrated as the ratio of the cumulative deficits present to the number of total deficits considered. Variables included in the frailty index model and the related values can be found in Table S3.

### Mortality outcomes

NHANES Public-Use Linked Mortality Files were used to determine the survival status of participants (as of December 31, 2019)^37^. The International Classification of Diseases, Tenth Revision (ICD-10) was used to define cause-specific death^38^. We examined all-cause death and cause-specific death, including cardiac diseases (ICD-10: I00-I09, I11, I13, I20-I51) and malignant neoplasms (ICD-10: C00-C97). The baseline for NHANES data collection was the starting point for calculating survival time.

### Covariates

The in-home questionnaire obtained information on age, sex, body mass index (BMI), race, education level, poverty income ratio, smoking status, drinking status, leisure-time physical activity, and type of cancer. Race was categorized into non-Hispanic white, black and other. Alcohol consumption in the past 12 months was defined as heavy drinking (≥ 2 drinks/day), mild drinking (1 drink/day), and nondrinking (no drinks). Cigarette consumption was defined as never having smoked (< 100 cigarettes in a lifetime), formerly smoking (≥ 100 cigarettes in a lifetime but has quit), and currently smoking (≥ 100 cigarettes in a lifetime and smokes some days or every day). Physical activity in leisure time was categorized into no or unable to engage in activity, moderate activity, and vigorous activity. The family poverty income ratio was equal to the family income divided by the poverty guideline, which corresponded to the year and the state of the participants^39^. Body mass index (BMI, kg/m^2^) was equal to weight (kg) divided by height (m) squared.

### Statistical analysis

Statistical analysis was performed with R (version 4.0.3). Two-sided P values below 0.05 were considered statistically significant. The weighted mean ± standard error (SE) are used for continuous variables, and counts (weighted frequencies) are used for categorical variables. Kaplan‒Meier survival analyses and Cox proportional hazards models were used to assess the association of frailty scores with all-cause and cause-specific mortality. Three statistical models were fitted. In Model 1, we adjusted for age (continuous), race (non-Hispanic white, non-Hispanic black, or others), and sex (male or female). In Model 2, we further adjusted for BMI (< 20, 20–24, 25–29, or ≥ 30 kg/m^2^), educational attainment (below high school, high school, or college or above), alcohol consumption (none, mild, or heavy), cigarette consumption (never, former, or current), poverty income ratio (≤ 1, 1–3, or > 3), and leisure-time physical activity (no, yes). In Model 3, we further adjusted for diabetes (no or yes), hyperlipidemia (no or yes), hypertension (no or yes), and the type of cancer (skin and soft tissue, urinary system, breast, genital system, digestive system, other). The linear trend of coefficients was examined by assigning a median value to each category as a continuous variable.

A restricted cubic spline model was employed to estimate and visualize the dose‒response relation between the frailty score and mortality risk with four knots determined at the 5th, 35th, 65th, and 95th percentiles^40^. Nonlinearity was assessed with likelihood ratio tests comparing models with and without the cubic spline term. Stratified analyses were performed by age (≤ 65, > 65 y), sex (male, female), race (white, other), BMI (< 30, ≥ 30), and type of cancer. The P values for the product terms between frailty score and stratification variables were used to test the significance of interactions.

Several sensitivity analyses were also performed to test the robustness of our findings. First, to avoid the potential effect of outliers, we refitted a cubic spline model between the frailty score (0.078 to 0.469) and mortality risk after excluding survivors with frailty scores outside the 5th and 95th percentiles (a total of 482 participants). Second, Kaplan‒Meier survival analyses were employed to examine the association of frailty score with other cause-specific mortality and residual mortality. Third, to account for the impact of specific cancer types on mortality, we reassessed the association between frailty and poor outcomes when additionally adjusting for detailed cancer classifications (29 types).

### Ethics disclosures

The NHANES was approved by the National Center for Health Statistics (NCHS) Research Ethics Review Board (https://www.cdc.gov/nchs/nhanes/irba98.htm).

### Supplementary Information

Supplementary Information.

## Data Availability

All data included in this study are available in the references section.
