# Supplementary material for: Association of frailty with mortality in cancer survivors: results from NHANES 1999–2018
Source: Sci Rep. 2024 Jan 18;14:1619. doi: 10.1038/s41598-023-50019-1 (PMC10796930; doi:10.1038/s41598-023-50019-1)

**Supplementary Materials**

**Tables**

**Table S1.** Detailed description of cancer classification

**Table S2.** Definition and proportion of cause of death

**Table S3.** Frailty index variables and scoring

**Table S4.** HRs (95% CIs) for other cause-specific mortality and residual mortality according to frailty score among patients with cancer

**Table S5.** HR (95% CI) for all-cause and cause-specific mortality according to frailty score with additional adjustment for detailed cancer classification (29 types)

**Figures**

**Figure S1.** Subgroup analyses of frailty score with cancer-specific mortality among patients with cancer.

**Figure S2.** Subgroup analyses of frailty score with cardiac-specific mortality among patients with cancer.

**Figure S3.** The dose-response association of frailty score with all-cause mortality (A), cancer mortality (B), and cardiac mortality (C) among patients with cancer.

**Figure S4.** Kaplan-Meier survival curve of other cause-specific mortality (A), residual mortality (B) according to frailty score tertiles among patients with cancer.

**Table S1.** Detailed description of cancer classification

| Classification | Detailed Location | Count |
| --- | --- | --- |
| Skin & Soft Tissue | Skin (non-melanoma) | 750 |
|  | Melanoma | 291 |
|  | Mouth/tongue/lip | 30 |
|  | Soft tissue (muscle or fat) | 12 |
| Urinary System | Prostate | 783 |
|  | Bladder | 120 |
|  | Kidney | 93 |
| Breast | Breast | 792 |
| Genital System | Cervix (cervical) | 327 |
|  | Uterus (uterine) | 208 |
|  | Ovary (ovarian) | 113 |
|  | Testis (testicular) | 30 |
| Digestive System | Colon | 346 |
|  | Stomach | 42 |
|  | Esophagus (esophageal) | 28 |
|  | Liver | 24 |
|  | Rectum (rectal) | 22 |
|  | Pancreas (pancreatic) | 11 |
|  | Gallbladder | 2 |
| Others | Other | 222 |
|  | Lung | 133 |
|  | Lymphoma/Hodgkin's disease | 108 |
|  | Thyroid | 96 |
|  | Leukemia | 51 |
|  | Bone | 31 |
|  | Larynx/ windpipe | 25 |
|  | Brain | 21 |
|  | Blood | 11 |
|  | Nervous system | 1 |

**Table S2.** Definition and proportion of cause of death

| Cause of death (ICD-10 code) | Count | Percent (%) |
| --- | --- | --- |
| Malignant neoplasms (C00-C97) | 581 | 32.7 |
| All other causes (residual) | 413 | 23.3 |
| Diseases of heart (I00-I09, I11, I13, I20-I51) | 385 | 21.7 |
| Chronic lower respiratory diseases (J40-J47) | 90 | 5.1 |
| Cerebrovascular diseases (I60-I69) | 84 | 4.7 |
| Alzheimer's disease (G30) | 70 | 3.9 |
| Accidents (unintentional injuries) (V01-X59, Y85-Y86) | 41 | 2.3 |
| Diabetes mellitus (E10-E14) | 39 | 2.3 |
| Influenza and pneumonia (J09-J18) | 36 | 2 |
| Nephritis, nephrotic syndrome and nephrosis (N00-N07, N17-N19, N25-N27) | 36 | 2 |
| Total | 1775 | 100 |

**Table S3. Frailty index variables and scoring**

| Item | | Variable in NHANES | Code |
| --- | --- | --- | --- |
| **Cognition** | |  |  |
|  | 1. experience confusion/memory problems | pfq056, pfq057 | yes=1; no=0 |
| **Dependence** | |  |  |
|  | 2. managing money difficulty | pfq060a, pfq061a | no difficulty=0; |
|  |  |  | some difficulty=0.33; |
|  |  |  | much difficulty=0.66; |
|  |  |  | unable to do=1 |
|  | 3. walking for a quarter mile difficulty | pfq060b, pfq061b | the same to above |
|  | 4. walking up ten steps difficulty | pfq060c, pfq061c | the same to above |
|  | 5. stooping, crouching, kneeling difficulty | pfq060d, pfq061d | the same to above |
|  | 6. lifting or carrying difficulty | pfq060e, pfq061e | the same to above |
|  | 7. house chore difficulty | pfq060f, pfq061f | the same to above |
|  | 8. preparing meals difficulty | pfq060g, pfq061g | the same to above |
|  | 9. walking between rooms on same floor | pfq060h, pfq061h | the same to above |
|  | 10. standing up from armless chair difficulty | pfq060i, pfq061i | the same to above |
|  | 11. getting in and out of bed difficulty | pfq060j, pfq061j | the same to above |
|  | 12. using fork, knife, drinking from cup difficulty | pfq060k, pfq061k | the same to above |
|  | 13. dressing yourself difficulty | pfq060l, pfq061l | the same to above |
|  | 14. standing for long periods difficulty | pfq060m, pfq061m | the same to above |
|  | 15. Sitting for long periods difficulty | pfq060n, pfq061n | the same to above |
|  | 16. reaching up over head difficulty | pfq060o, pfq061o | the same to above |
|  | 17. grasp/holding small objects difficulty | pfq060p, pfq061p | the same to above |
|  | 18. going out to movies, events difficulty | pfq060q, pfq061q | the same to above |
|  | 19. attending social event difficulty | pfq060r, pfq061r | the same to above |
|  | 20. leisure activity at home difficulty | pfq060s, pfq061s | the same to above |
|  | 21. push or pull large objects difficulty | pfq061t | the same to above |
| **Depressive Symptoms** | |  |  |
|  | 22. have little interest in doing things | ciqd008, ciqd009, dpq010 | ~2003 |
|  |  |  | every day, nearly every day = 1 |
|  |  |  | most days = 0.75 |
|  |  |  | about half the days = 0.50 |
|  |  |  | less than half the days = 0.25 |
|  |  |  | 2005~ |
|  |  |  | nearly every day = 1 |
|  |  |  | more than half the days = 0.66 |
|  |  |  | several days = 0.33 |
|  | 23. feeling down, depressed, or hopeless | dpq020, ciqd001, ciqd002 | the same to above |
|  | 24. trouble sleeping or sleeping too much | dpq030, ciqd025, ciqd026 | ~2003 |
|  |  |  | every night = 1 |
|  |  |  | nearly every night = 0.66 |
|  |  |  | less often = 0.33 |
|  |  |  | 2005~ |
|  |  |  | nearly every day = 1 |
|  |  |  | more than half the days = 0.66 |
|  |  |  | several days = 0.33 |
|  | 25. feeling tired or having little energy | dpq040 | nearly every day = 1 |
|  |  |  | more than half the days = 0.66 |
|  |  |  | several days = 0.33 |
|  | 26. poor appetite or overeating | ciqd019, ciqd022, dpq050 | ~2003 |
|  |  |  | yes = 1 |
|  |  |  | no = 0 |
|  |  |  | 2005~ |
|  |  |  | the same to above |
|  | 27. feeling bad about yourself | dpq060, ciqd029 | the same to above |
|  | 28. trouble concentrating on things | dpq070, ciqd043 | the same to above |
| **Comorbidities** | |  |  |
|  | 29. doctor ever said you had arthritis | mcq160a | yes = 1; no = 0 |
|  | 30. ever told you had thyroid problem | mcq160i, mcd160m, mcq160m | the same to above |
|  | 31. ever told you had chronic bronchitis | mcq160k, mcq160p | the same to above |
|  | 32. ever told you had cancer or malignancy | mcq220 | the same to above |
|  | 33. ever told had congestive heart failure | mcq160b | the same to above |
|  | 34. ever told you had coronary heart disease | mcq160c | the same to above |
|  | 35. ever told you had angina/angina pectoris | mcq160d | the same to above |
|  | 36. ever told you had heart attack | mcq160e | the same to above |
|  | 37. ever told you had a stroke | mcq160f | the same to above |
|  | 38. ever told you had high blood pressure | bpq020 | the same to above |
|  | 39. doctor told you have diabetes | diq010 | yes = 1; no =0; borderline=0.5 |
|  | 40. ever told you had weak/failing kidneys | kiq020, kiq022 | yes = 1; no =0 |
|  | 41. urine leakage bother you? | kiq040, kiq050 | 1999 |
|  |  |  | yes = 1; no = 0 |
|  |  |  | 2001~ |
|  |  |  | greatly = 1 |
|  |  |  | very much = 0.75 |
|  |  |  | somewhat = 0.5 |
|  |  |  | only a little = 0.25 |
| **Hospital Utilization and Access to Care** | |  |  |
|  | 42. general health condition | huq010 | excellent,very good,good = 0 |
|  |  |  | fair, poor = 1 |
|  | 43. health now compared with 1 year ago | huq020 | about the same, better = 0 |
|  |  |  | worse = 1 |
|  | 44. overnight hospital patient in last year | huq070, hud070, huq071 | yes = 1, no = 0 |
|  | 45. times receive healthcare over past year | huq050, huq051 | none = 0; 1-4 = 0.5; >=5 =1 |
|  | 46. number of prescription medicines taken | rxd030, rxduse, rxd295, rxdcount | no = 0; 1-4 = 0.5; >=5 =1 |
| **Physical Performance and Anthropometry** | | - | - |
|  | 47. body mass index (kg/m^2) | bmxbmi | <18.5, ≥30 = 1 |
|  |  |  | 25–<30 = 0.5 |
|  |  |  | 18.5–25 = 0 |
| **Laboratory Values** | |  |  |
|  | 48. glycohemoglobin (%) | lbxgh | 0%–5.7% = 0, >5.7% = 1 |
|  | 49. red blood cell count (million cells/ul) | lbxrbcsi | Male: 4.7–6.1 = 0, Other = 1 |
|  |  |  | Female: 4.2–5.4 = 0, Other = 1 |
|  | 50. hemoglobin (g/dl) | lbxhgb | Male: 13.5–18 = 0, Other = 1 |
|  |  |  | Female: 12–16 = 0, Other = 1 |
|  | 51. red cell distribution width (%) | lbxrdw | 11.6–14.6 = 0, Other = 1 |
|  | 52. lymphocyte percent (%) | lbxlypct | 20–40 = 0, Other = 1 |
|  | 53. segmented neutrophils percent (%) | lbxnepct | 40–80 = 0, Other = 1 |

**Table S4.** HRs (95% CIs) for other cause-specific mortality and residual mortality according to frailty score among patients with cancer

| Characteristics | Frailty score | | | P-trend | Per-unit increment of frailty score (Ln-transformed) |
| --- | --- | --- | --- | --- | --- |
|  | Tertile 1 [0.0345,0.152) | Tertile 2 [0.1516,0.240) | Tertile 3 [0.2403,0.767] |  |  |
| Other cause-specific mortality | |  |  |  |  |
| Model 1 | 1 (reference) | 1.516 (1.012, 2.271) | 2.786 (1.926, 4.031) | <0.001 | 2.452 (1.767, 3.403) |
| Model 2 | 1 (reference) | 1.442 (0.958, 2.169) | 2.403 (1.636, 3.529) | <0.001 | 2.147 (1.537, 2.998) |
| Model 3 | 1 (reference) | 1.35 (0.882, 2.067) | 2.137 (1.419, 3.219) | <0.001 | 1.955 (1.366, 2.798) |
| Residual mortality |  |  |  |  |  |
| Model 1 | 1 (reference) | 1.691 (1.203, 2.379) | 3.899 (2.808, 5.414) | <0.001 | 3.718 (2.848, 4.853) |
| Model 2 | 1 (reference) | 1.674 (1.162, 2.411) | 3.563 (2.463, 5.157) | <0.001 | 3.473 (2.589, 4.659) |
| Model 3 | 1 (reference) | 1.627 (1.107, 2.391) | 3.528 (2.393, 5.199) | <0.001 | 3.691 (2.708, 5.032) |

HRs (95% CIs) were estimated by Cox proportional hazards model and accounted for the sample weights. Note: Residual mortality is referred to NHANES ICD10 undefined deaths. Other cause-specific mortality is defined as specific cause of mortality other than cardiac-specific mortality, cancer-specific mortality and residual mortality (referred to Supplement Table 1). Model 1 was adjusted for age (continuous), race (non-Hispanic white, non-Hispanic black, or others), and gender (male or female). Model 2 was additionally adjusted for BMI (<20, 20-24, 25-29, or ≥30 kg/m^2^), educational attainment (below high school, high school, or college or above), alcohol consumption (none, mild, or heavy), cigarette consumption (never, former, or current), poverty income ratio (≤1, 1-3, or >3), and leisure-time physical activity (no, yes). Model 3 was additionally adjusted for diabetes (no or yes), hyperlipidemia (no or yes), hypertension (no or yes), the type of cancer (skin & soft tissue, urinary system, breast, genital system, digestive system, others).

**Table S5.** HR (95% CI) for all-cause and cause-specific mortality according to frailty score with additional adjustment for detailed cancer classification (29 types)

| Outcomes | Tertile 1 | Tertile 2 | Tertile 3 | P-trend | Per-unit increment of frailty score (Ln-transformed) |
| --- | --- | --- | --- | --- | --- |
| All-cause mortality | 1 (reference) | 1.453 (1.244, 1.699) | 2.725 (2.29, 3.243) | <0.001 | 2.525 (2.188, 2.912) |
| Cancer mortality | 1 (reference) | 1.147 (0.876, 1.503) | 2.164 (1.658, 2.825) | <0.001 | 2.071 (1.673, 2.563) |
| Cardiac mortality | 1 (reference) | 2.033 (1.446, 2.859) | 4.008 (2.852, 5.631) | <0.001 | 3.684 (2.784, 4.875) |

HR (95% CI) was estimated by Cox proportional hazards model and accounted for the sample weights. According to the ICD-10 criteria, cardiac mortality was defined as I00-I09, I11, I13, I20-I51, and cancer mortality was defined as C00-C97. Models were adjusted for age, race, gender, BMI, educational attainment, alcohol consumption, cigarette consumption, poverty income ratio, and leisure-time physical activity, diabetes, hyperlipidemia, hypertension, and the detailed cancer classification (29 types). See Table S1 for specific cancer classifications.

**Figure S1.** Subgroup analyses of frailty score with cancer-specific mortality among patients with cancer.


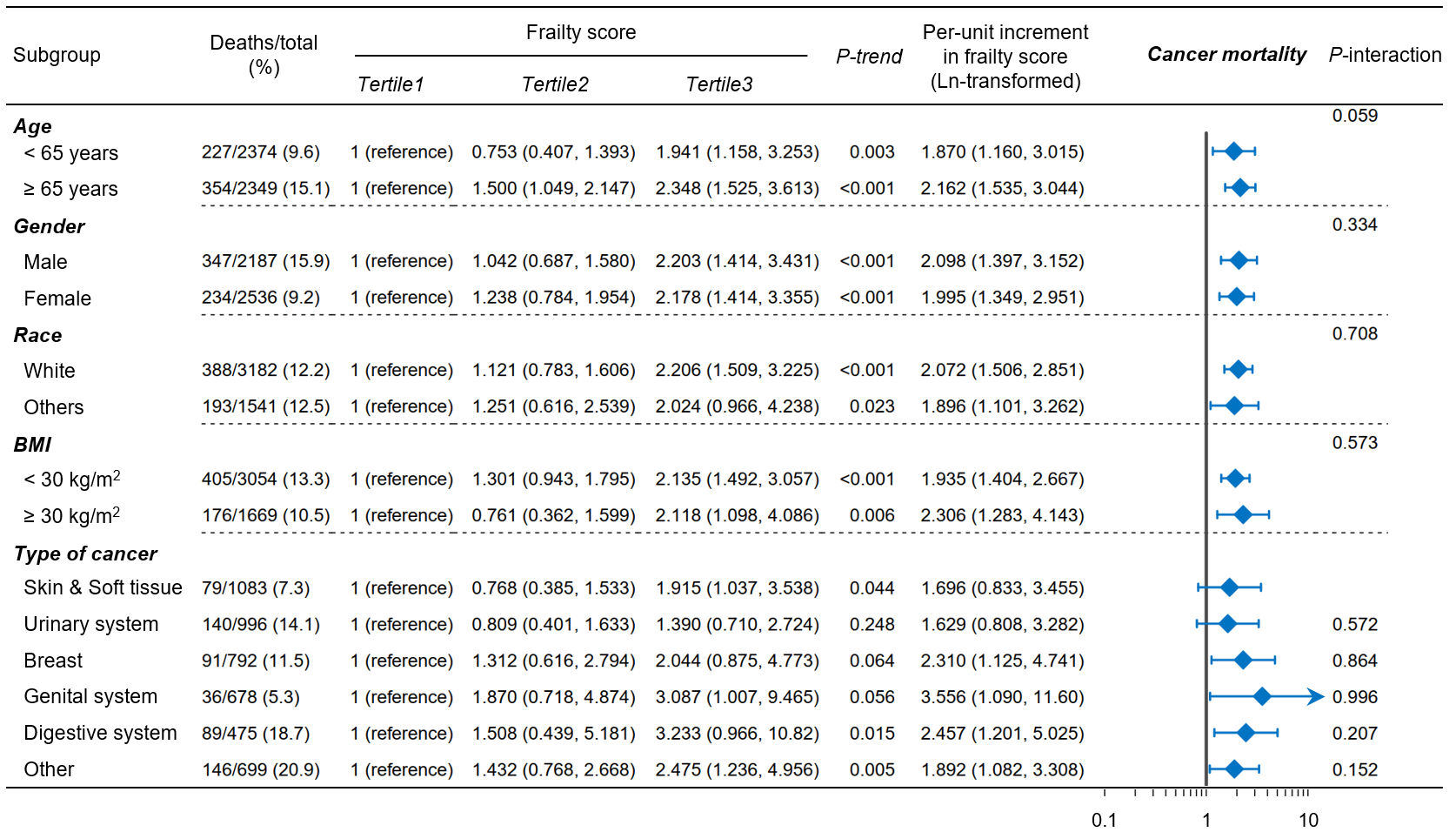


**Figure S2.** Subgroup analyses of frailty score with cardiac-specific mortality among patients with cancer.


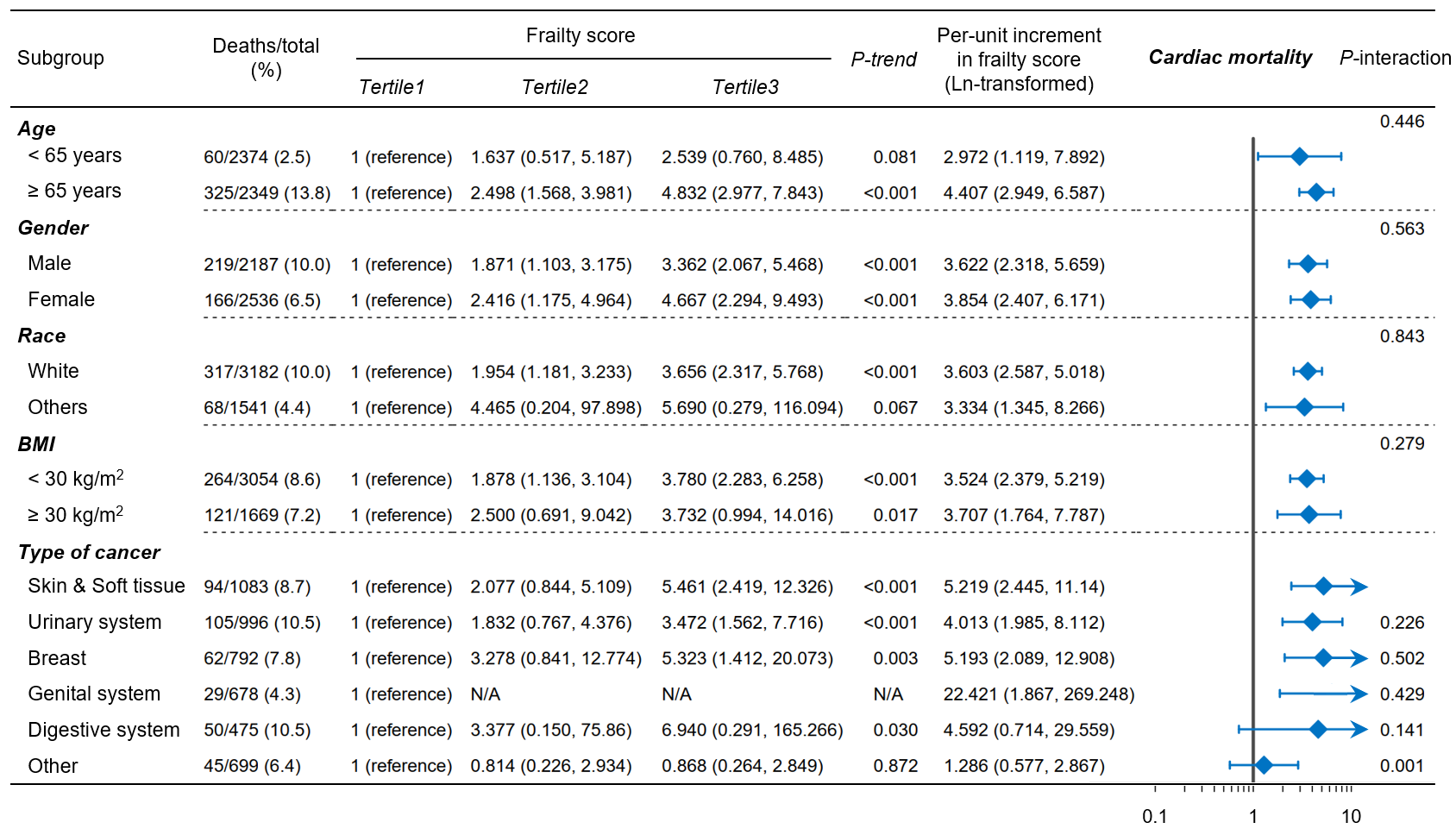


**Figure S3.** The dose-response association of frailty score with all-cause mortality (A), cancer mortality (B), and cardiac mortality (C) among patients with cancer.


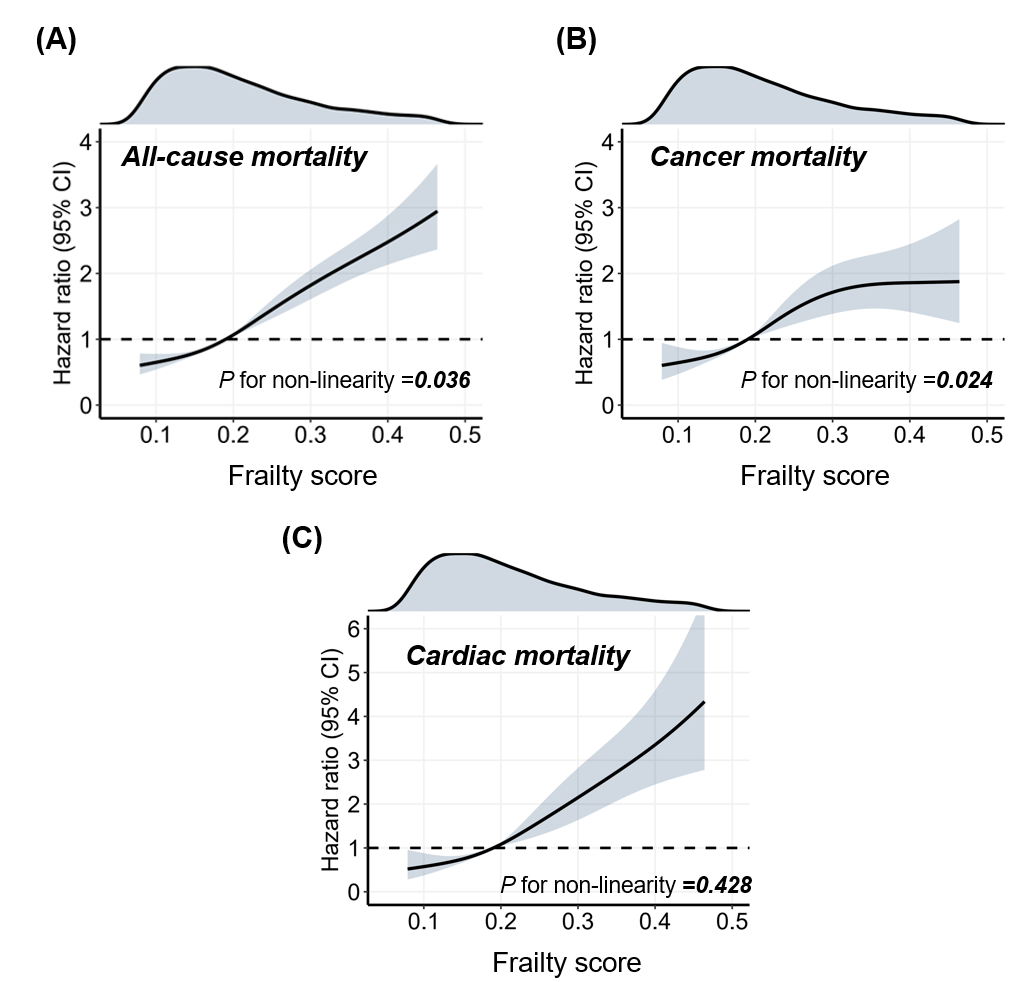


To avoid the potential effect of outliers, we excluded patients with frailty score outside the 5th and 95th percentile (excluding 482 samples). Then, a restricted cubic spline model was employed to visualize the relationship between frailty score (0.078-0.469 mg/day) and mortality risk with four knots determined at the 5th, 35th, 65th, and 95th percentiles.

**Figure S4.** Kaplan-Meier survival curve of other cause-specific mortality (A), residual mortality (B) according to frailty score tertiles among patients with cancer.


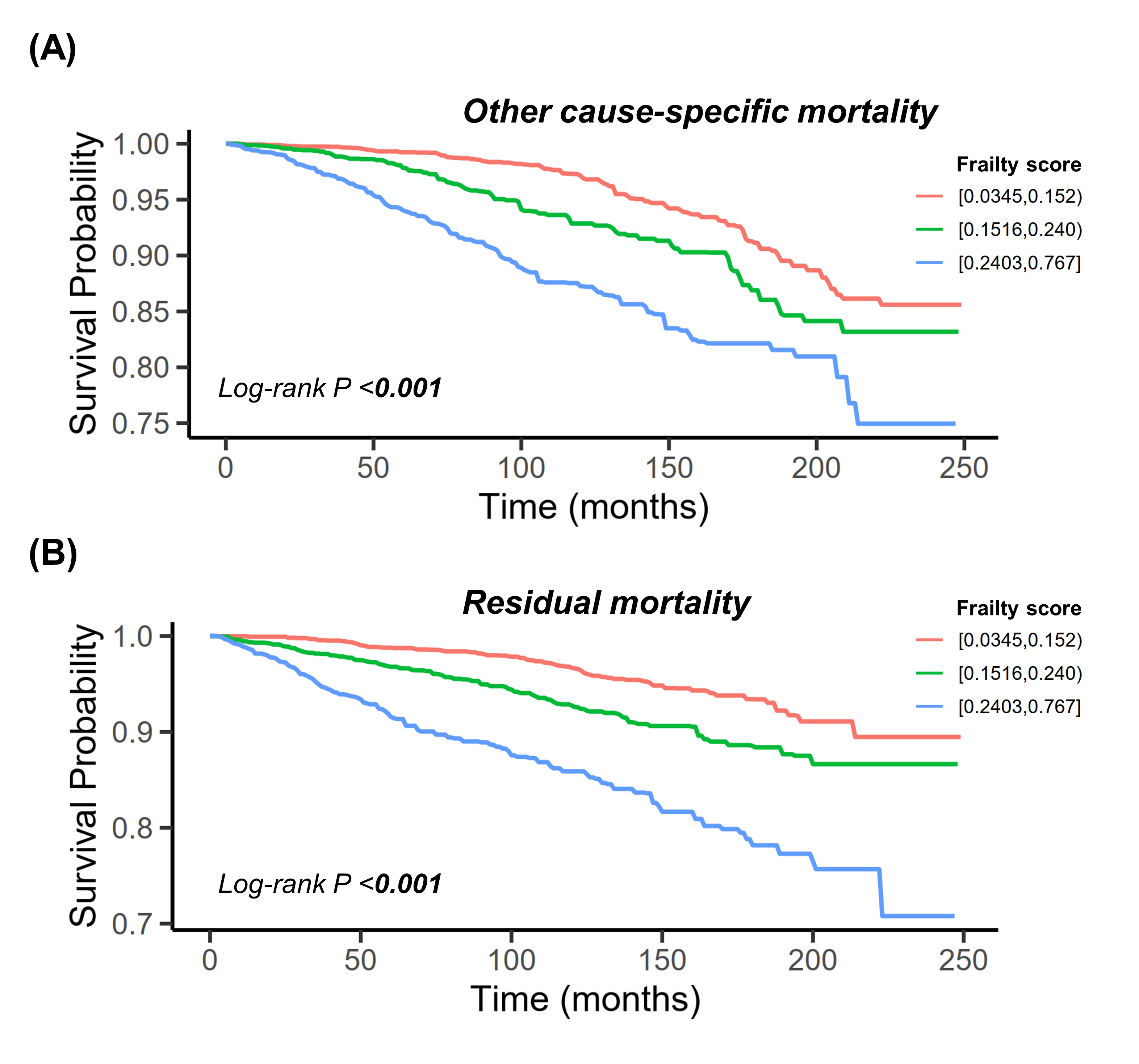

Supplement: Supplementary file 1 — Supplementary Information. [file 41598_2023_50019_MOESM1_ESM.doc]
